# Caecal diverticulitis presenting as acute appendicitis: a case report

**DOI:** 10.1186/1749-7922-4-29

**Published:** 2009-07-31

**Authors:** Michelle Cole, Abraham A Ayantunde, John Payne

**Affiliations:** 1 Department of Surgery, Queen Mary's Hospital, Frognal Avenue, Sidcup, Kent, DA14 6LT, UK

## Abstract

Solitary caecal diverticulum is an uncommon entity and therefore difficult to diagnose except at surgery. Caecal diverticulitis is an infrequent cause of acute abdomen and usually presents in a manner similar to acute appendicitis. It is extremely difficult to differentiate it preoperative from acute appendicitis and such distinction is usually made in the operating room. The optimal management of this clinical condition is still controversial, ranging from conservative treatment with antibiotics to aggressive surgical resections.

We report a case of a 61 year old Caucasian who presented with acute onset right iliac fossa pain indistinguishable from acute appendicitis. The true diagnosis of a perforated acute caecal diverticulitis with an abscess mass was only made at operation in the presence of a macroscopically normal appendix. We reviewed the literature to highlight the difficulty of a preoperative diagnosis and the need for a high index of suspicion especially in the older age group presenting in manner similar to acute appendicitis.

## Background

Solitary caecal diverticulum is an uncommon entity and therefore difficult to diagnose except at surgery. It is rare in the Western world among the Caucasians but has been shown to have a high incidence in the people of Asian origin or Oriental populations [1,2]. Caecal diverticulum is an infrequent cause of acute abdomen and caecal diverticulitis usually presents in a manner similar to acute appendicitis [3]. It is extremely difficult to differentiate it preoperative from acute appendicitis and such distinction is usually made in the operating room [4]. It is sometimes confused with caecal pole tumour when it presents with a right iliac fossa mass in the older age group [5]. There have been various debates in the literature about the most appropriate and optimal management of symptomatic solitary caecal diverticulum or caecal diverticulitis. Some studies have suggested a conservative approach, a wedge resection of the diverticulum, right hemicolectomy or ileo-caecal resection [1-4,6].

We report a case of solitary caecal diveticulitis presenting as an acute appendicitis to highlight the dilemma in preoperative diagnosis and present the review of the literature on the investigations and management debates and diversity.

## Case report

A 61 year old Caucasian man presented to our Accident and Emergency unit with a day history of right iliac fossa pain associated with fever and rigors. The appetite was reduced but no nausea or vomiting. The pain was said to be constant and sharp in nature and exacerbated by movement and stretching. He denies any history of a recent altered bowel habit or urinary symptoms. The only significant past medical history were renal calculi and well controlled asthma.

Physical examination revealed mild dehydration and normal vital signs. His abdomen was full with tenderness in the right iliac fossa and associated with guarding and local peritonitis. Blood investigations showed haemoglobin level of 14.0 g/dl, total white blood cell count of 22.4 with neutrophilia of 20.0, platelet count of 326, C-reactive protein of 36 and normal electrolytes, urea, amylase and liver function tests.

The initial diagnosis of acute appendicitis was entertained and patient was prepared and consented for an open appendicectomy. The findings at operation included a 4 cm by 5 cm pericaecal abscess mass adjacent to the anterior tenia coli. Within the abscess mass was a perforated anterior caecal diverticulum with necrotic wall. There was a polypoid mass within the wall of the caecum. The appendix was macroscopically normal with no evidence of acute inflammation. There was a suspicion of a perforated caecal tumour. He then underwent a right hemicolectomy with an ileo-transverse anastomosis through a medial extension of the appendicectomy wound.

**Figure 1:**
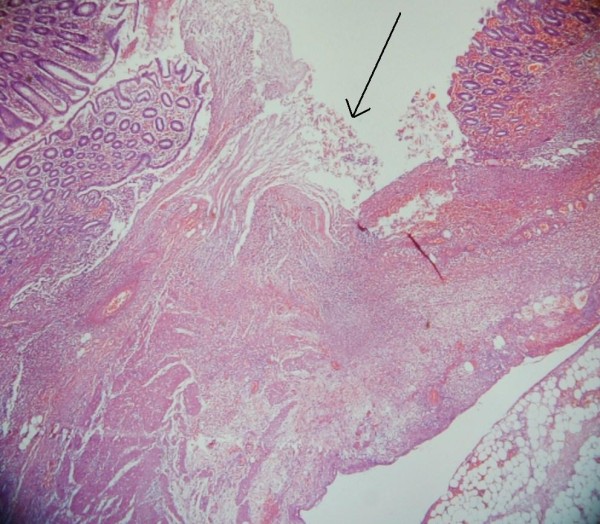
**Showing partially maintained diverticulum mucosal lining with erosion and loose granulation tissues with acutely inflamed serosa and extramural fat (indicated with black arrow)**.

**Figure 2:**
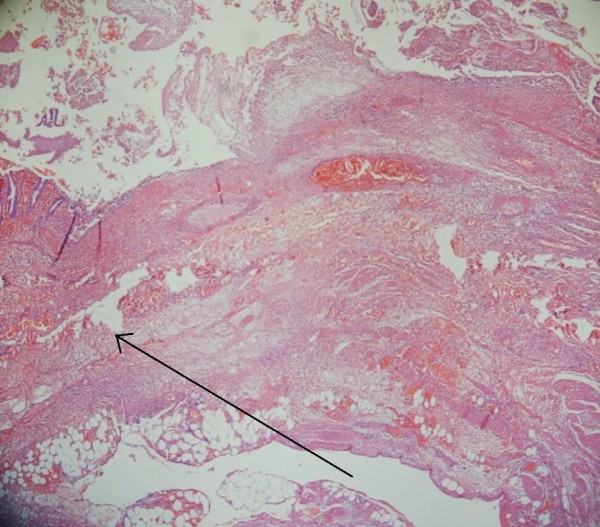
**The perforation of the diverticulum mucosal is extending into the extramural fat (indicated with black arrow)**.

The histology of the right hemicolectomy specimen macroscopically showed an inflamed and perforated solitary caecal diverticulum with abscess formation and an isolated caecal pedunculated polyp. Microscopically no dysplasia or malignancy within the caecal diverticulum and the polyp was a tubulovillous adenoma with low grade dysplasia. The caecal diverticulum lacked mucularis propria and therefore was considered to be acquired [Figures 1 and 2].

His postoperative course was uneventful and he was discharged home within a week of admission with an outpatient colonoscopy planned to evaluate the rest of his bowel. His follow up colonoscopy revealed further left sided colonic polyps with histology showing tubulovillous adenoma with moderate dysplasia.

## Discussion

Solitary caecal diverticulum is uncommon and the first description in literature was by Potier in 1912 [1,3]. Several cases have been reported since its first description but its preoperative diagnosis continues to be very elusive. The reported frequency in literature has been estimated to be 1 in 300 appendicectomies [4,7]. It accounts for 3.6% of all colonic diverticula with median age incidence of 44 years and male to female ratio of 3:2 [8]. Caecal diverticulitis is a rare cause of right iliac fossa abdominal pain in Caucasian patients, but is rather more common amongst the Asian or Oriental populations [1,2]. It usually presents in a manner similar to an acute appendicitis and the two are clearly indistinguishable except occasionally by imaging investigations but mostly at operation [3-5]. Lane et al [6] reported that more than 70% of patients with caecal diverticulitis were operated on with a presumptive preoperative diagnosis of acute appendicitis.

Solitary caecal diverticulum has been classified into congenital (true) and acquired (false) groups. Congenital or true diverticulum contains all layers of the colonic wall and embryologically is thought to have arisen from a transient outpouching of the caecal wall at about 6 weeks of gestational age. This outpouching usually atrophies at about seventh week gestation but occasionally may fail to regress and presents an impression of duplicated appendix [5,6,9,10]. However, an acquired counterpart is considered a false diverticulum arising from similar aetiopathogenetic factors as the left sided diverticular disease and therefore does not contain muscular wall layer [10]. The majority of caecal diverticula arise from the anterior aspect of the caecum and usually solitary, thus when inflamed they have the tendency to perforate and cause peritionitis. However, a posteriorly situated caecal diverticulitis when perforated may present as a caecal mass and mimicking a perforated carcinoma [5].

The preoperative diagnosis of caecal diverticulitis is difficult and one report claims it is only made in 9% of the cases, and most of these patients have had previous appendicectomy [5]. Acute appendicitis is by far the initial preoperative diagnosis on account of similar presentation of low grade fever, right iliac fossa pain and tenderness with guarding. However some studies have suggested certain subtle clinical features that may help in differentiating caecal diverticulitis from acute appendicitis including a relatively longer history of right iliac fossa pain with relative lack of systemic toxicity despite prolonged duration of the symptoms especially with a posteriorly located lesion. Nausea and vomiting are less frequent, abdominal pain typically starts and remains in the right iliac fossa rather than beginning in the central abdomen and shifting to the RIF and RIF tenderness is not usually as marked as in acute appendicitis [2,5,11].

As acute appendicitis is mainly a clinical diagnosis, many patients presenting with RIF pain and tenderness with a presumptive diagnosis of appendicitis are usually not subjected to preoperative radiological investigations except where the diagnosis is in doubt especially in female patients or where a mass is palpable and a caecal carcinoma is suspected. Our patient did not have a preoperative radiological investigation because of a strong suspicion of an acute appendicitis. However, abdominal ultrasound scan (USS) and computerised tomography (CT) scan have been shown to have high diagnostic accuracy and may play a role in the preoperative diagnosis [5,12-14]. In a review of 934 patients with caecal diverticulitis presenting with indeterminate right lower quadrant abdominal pain, Chou et al [13] reported a sensitivity of 91.3% and a specificity of 99.5% with abdominal USS with an overall accuracy of 99.5% in the diagnosis of caecal diverticulitis. The preoperative accuracy of distinguishing acute appendicitis from caecal diverticulitis with CT scan has been shown in various studies with a sensitivity and specificity of 98% [8,12,13]. The CT scan findings are similar to those of left sided diverticulitis, including caecal wall thickening, focal pericaecal inflammation with stranding of adjacent fascia, associated diverticulitis, abscess formation, extraluminal air signifying perforation and extraluminal mass effect [8,12,13,15]. These features may however be present with caecal pole tumour especially in the presence of tumour perforation or associated peritumoural inflammatory reaction. Caecal diverticulitis may actually be indistinguishable from carcinoma on the CT scan in about 10% of cases [15].

The early use of diagnostic laparoscopic in lower abdominal pain of indeterminate cause may be a useful tool in allowing accurate diagnosis and planning the appropriate treatment. This is particularly important especially in young women with possible gynaecological pathology as the cause of the pain. The surgical management of non-perforated caecal diverticulitis is highly controversial unlike that of the left sided diverticulitis [1-4,6,7]. Conservative management with intravenous antibiotics and supportive care are the rule when a preoperative diagnosis of non-perforated caecal diverticulitis is made with confidence [3,14,15]. Conservative management can still be pursued even after an accurate diagnosis of uncomplicated caecal diverticulitis is made at diagnostic laparoscopy after an adequate washout. Complicated caecal diverticulitis with perforation can also be managed laparoscopically if the expertise is available [9,16].

The variation in surgical resection ranges from an isolated diverticulectomy, ileocaecal resection or standard right hemicolectomy. Laparoscopic diverticulectomy has been described in the management of right-sided diverticulitis [16]. Fang et al [7] have recommended an aggressive resection in a review of 85 cases in an Asian population. Successful resolution of diverticulitis was achieved in less than 40% of their cases and this outcome informed their recommendation. Lane et al [6] in another series of 49 patients reported that 40% of their patients treated with diverticulectomy or antibiotics alone required subsequent hemicolectomy due to an on-going inflammatory process. Right hemicolectomy carries a higher morbidity and mortality but is generally recommended for an inflammatory mass where diverticulectomy is usually impossible or when a carcinoma is suspected and an adequate lymphovascular clearance should be performed in such cases [5-7,14,15]. Our patient underwent a right hemicolectomy and standard lymphovascular clearance because of the findings of inflammatory mass in the presence of a normal appendix and a polypoid mass within the caecum.

## Conclusion

Solitary caecal diverticulum is rare especially in the Caucasian populations. Therefore, a high index of suspicion is required in the diagnosis of caecal diverticulitis and should be considered as a possible differential diagnosis in patients presenting with acute right iliac fossa pain. Its diagnosis should particularly be suspected in patients with a longer history of pain with atypical presentations of acute appendicitis. Generally, this clinical condition is mostly diagnosed intraoperatively but the use of abdominal USS or CT scan may lead to preoperative diagnosis. One needs to develop a low threshold for the use of a diagnostic laparoscopy in patients and especially in women with atypical presentations of acute appendicitis. An uncomplicated caecal diverticulitis, when a preoperative diagnosis is made convincingly should be managed conservatively with intravenous antibiotics. However, majority of the cases are treated surgically because of difficulty distinguishing it from an acute appendicitis or excluding a caecal carcinoma. There are different surgical approaches and generally, a right hemicolectomy is recommended in the presence of an inflammatory mass and when a carcinoma cannot be excluded.

## Consent

Written informed consent was obtained from the patient for publication of this case report and any accompanying images. A copy of the written consent is available for review by the Editor-in-Chief of this journal.

## Abbreviations

CT: scan, computerised tomography scan; RIF: right iliac fossa; USS: ultrasound scan;

## Competing interests

The authors declare that they have no competing interests.

## Authors' contributions

MC participated in the admission and the care of this patient, the conception, the design, data collection and interpretation, manuscript preparation and literature search. AAA participated in the admission and the care of this patient, the conception, the design, data collection and interpretation, manuscript preparation and literature search. JP participated in the admission and the care of this patient, the conception, the design, data collection and interpretation, manuscript preparation and literature search. All authors read and approved the final manuscript.
